# Analysis of characteristics and outcomes by growth hormone treatment duration in adult patients in the Italian cohort of the Hypopituitary Control and Complications Study (HypoCCS)

**DOI:** 10.1007/s40618-018-0860-x

**Published:** 2018-03-13

**Authors:** V. Rochira, G. Mossetto, N. Jia, S. Cannavo, P. Beck-Peccoz, G. Aimaretti, M. R. Ambrosio, C. Di Somma, M. Losa, D. Ferone, C. Lubrano, C. Scaroni, A. Giampietro, S. M. Corsello, M. Poggi

**Affiliations:** 1 Unit of Endocrinology, Department of Biomedical, Metabolic and Neural Sciences, University of Modena and Reggio Emilia, Azienda Ospedaliero-Universitaria di Modena, Ospedale Civile di Baggiovara, Via Pietro Giardini 1355, 41126 Modena, Italy; 2 Eli Lilly, Sesto Fiorentino, Italy; 3 Eli Lilly, Indianapolis, IN USA; 4 University of Messina, Messina, Italy; 5 IRCCS Policlinico, University of Milan, Milan, Italy; 6 University of Eastern Piedmont, Novara, Italy; 7 University of Ferrara, Ferrara, Italy; 8 IRCCS SDN, Naples, Italy; 9 IRCCS San Raffaele Hospital, Milan, Italy; 10 DiMI, IRCCS AOU San Martino-IST University of Genova, Genoa, Italy; 11 La Sapienza University, Policlinico Umberto I, Rome, Italy; 12 Medical Sciences DIMED, University of Padova, Padua, Italy; 13 Università Cattolica del S. Cuore, Rome, Italy; 14 St. Andrea Hospital, Rome, Italy

**Keywords:** Growth hormone deficiency, Growth hormone treatment, Adult, GH dose, Insulin-like growth factor-I, Lipid profile

## Abstract

**Purpose:**

To examine differences in effects according to growth hormone (GH) treatment duration in adult GH-deficient patients.

**Methods:**

In the Italian cohort of the observational Hypopituitary Control and Complications Study, GH-treated adults with GH deficiency (GHD) were grouped by duration of treatment; ≤ 2 years (*n *= 451), > 2 to ≤ 6 years (*n *= 387) and > 6 years (*n *= 395). Between-group differences in demographics, medical history, physical characteristics, insulin-like growth factor-I standard deviation score (IGF-I SDS) and lipid profile at baseline, last study visit and changes from baseline to last study visit were assessed overall, for adult- and childhood-onset GHD and by gender using ANOVA for continuous variables and Chi-squared test for categorical variables.

**Results:**

At baseline, treatment duration groups did not differ significantly for age, gender, body mass index, GHD onset, IGF-I SDS, lipid profile, and quality of life. Mean initial GH dose did not differ significantly according to treatment duration group in any subgroup, except female patients, with highest mean dose seen in the longest duration group. In the longest duration group for patients overall, adult-onset patients and male patients, there were significant decreases in GH dose from baseline to last visit, and in total and low-density lipoprotein (LDL)-cholesterol concentrations. IGF-I SDS increased, to a greater extent, in the longest duration group for patients overall and female patients.

**Conclusions:**

The results show that long-term GH treatment is associated with decreasing GH dose, increased IGF-I, decreased LDL-cholesterol and the presence of surrogate markers that help to give confidence in a diagnosis of GHD.

## Introduction

Growth hormone (GH) deficiency in adult patients is associated with increased risk of mortality, predominantly due to cardiovascular disease [1–4]. GH-deficient patients frequently have excess body fat, decreased lean body mass and an abnormal lipid profile, and GH treatment is able to reverse these effects [2–8]. Adult patients diagnosed as having GH deficiency require continuous treatment with GH throughout their lives to maintain the metabolic benefits. However, the effects of such continuous GH treatment may change over time and data are required to evaluate such potential changes.

Data from large observational studies indicate that duration of GH treatment varies widely from a few months to many years. However, only limited data have been published that enable comparison of metabolic and hormonal effects between short-term versus long-term GH treatment [8, 9]. In the observational Hypopituitary Control and Complications Study (HypoCCS) programme, data have been collected for a large number of adult patients with GH deficiency who were treated with GH [10, 11]. Among the 16 countries in Europe and North America that enrolled patients into HypoCCS, Italy was a major contributing country to the database [12]. The present analysis used the Italian cohort of patients in HypoCCS, which included data from patients with various durations of GH treatment. The present analysis was carried out to examine differences in baseline characteristics, efficacy and quality of life between patients who received GH treatment for different durations, to investigate changes in effects during short-term versus long-term treatment.

## Methods

### Patients and study design

HypoCCS was a multi-national surveillance study designed to examine the safety and efficacy of long-term GH treatment in adult patients with GH deficiency. Patients entered into the study were adults, aged at least 18 years, with onset of GH deficiency either during childhood (CO) or adulthood (AO). Because HypoCCS was a surveillance study, the diagnosis of GH deficiency, entry into the study and all treatment decisions were at the discretion of the attending physician according to local guidelines. The Italian cohort of patients who entered into the present analysis met the criteria for the indication of adult treatment with GH, according to Italian reimbursement rules described in Nota AIFA no. 39.

Other than a diagnosis of GH deficiency, the only inclusion criterion for entry to HypoCCS was that patients had achieved adult height, with closed epiphyses. Exclusion criteria included active malignancy, recent growth of a pituitary adenoma or other intracranial tumour, presence of acute critical illness due to complications following open heart or abdominal surgery, multiple accidental traumas, or acute respiratory failure.

HypoCCS was registered with ClinicalTrials.gov, study number NCT01088399. The clinical study protocol was approved by appropriate institutional review boards and adhered to all applicable regulatory requirements. All patients provided signed informed consent for data collection, electronic processing and publication, in accordance with national laws. The study was performed according to the ethical principles of the Declaration of Helsinki.

### Study evaluations

Data collected at baseline and during follow-up included demographics, medical history, height, weight, body mass index (BMI), insulin-like growth factor-I (IGF-I) concentrations, lipid profile and Framingham cardiovascular risk index. Study visits for clinical assessment occurred at approximately 6- to 12-month intervals, although time between visits was flexible, in line with clinical practice.

Peak GH concentration following stimulation testing was determined at local laboratories using commercially available GH assays. IGF-I concentration was measured at a central laboratory (IGF Labor, Universitats Kinderklinik, Giessen, Germany) and values were converted to standard deviation scores (SDS) based on age- and gender-matched reference ranges, using published methods [13]. Concentrations of total cholesterol, low-density lipoprotein (LDL)-cholesterol, high-density lipoprotein (HDL)-cholesterol and triglycerides were assessed locally using the routine procedures of the hospital laboratories. The Framingham cardiovascular index was determined as 10-year risk according to published calculations [14]. The risk score was calculated as the sum of by-gender sub-scores of age, total cholesterol level, HDL-cholesterol level, smoking history and systolic blood pressure determined at each time point. Quality of life was evaluated from the Questions on Life Satisfaction-Hypopituitarism (QLS-H) questionnaire and *Z*-scores calculated from normative data [15].

### Data analysis and statistics

Data were analysed for patients in Italy who were followed in HypoCCS from 1995 to December 2012. Duration of GH treatment for these patients ranged from < 1 to > 16 years. For the present analysis, the patients were divided into three groups based on duration of therapy: group A received GH treatment for ≤ 2 years (*n *= 451), group B received GH for > 2 to ≤ 6 years (*n *= 387) and group C received GH for > 6 years (*n *= 395). The following parameters were evaluated: demographics, GH deficiency diagnosis, BMI, lipid profile, GH dose, IGF-I SDS, Framingham cardiovascular risk and quality of life. Data are shown as mean ± standard deviation or with 95% confidence intervals (CI) for continuous variables, and number of patients (percentage of total patient number) for categorical variables. Baseline variables and changes from baseline to last available visit on GH treatment were compared across treatment duration groups. Differences between the duration of treatment groups were assessed using ANOVA for continuous variables and Chi-squared tests for categorical variables, with *P *< 0.05 considered statistically significant. All analyses of differences between duration groups were repeated for subgroups based on the onset of GH deficiency (AO and CO), and gender (male and female). The effect of age at last visit was assessed for continuous variables using logistic regression with group C as the reference group, assessing effect of age and its interaction with treatment duration groups. No adjustments for multiplicity were made due to the ad hoc nature of the analysis.

## Results

There were no statistically significant differences between the duration-of-treatment groups for mean baseline age, proportion of males, BMI, onset of GH deficiency, lipid profile, IGF-I SDS or quality of life (Table 1). These parameters did not differ significantly when the groups were compared within individual subgroups of AO, CO, male and female patients. Peak GH following GH-releasing hormone (GHRH) + arginine tests did not differ significantly between duration groups overall or for any individual subgroup.

**Table 1:** Patient demographics and characteristics at baseline

	Group A (*N *= 451)	Group B (*N *= 387)	Group C (*N *= 395)	*P* value
Age at baseline (years)	44.1 ± 16.2	43.8 ± 16.3	43.2 ± 14.8	0.699
Gender, male *n* (%)	253 (56%)	235 (61%)	235 (60%)	0.365
Body mass index (kg/m^2^)	28.6 ± 7.3	28.0 ± 6.1	27.7 ± 5.6	0.133
GH deficiency onset				0.345
Adult onset *n* (%)	336 (75%)	303 (78%)	308 (78%)	
Childhood onset *n* (%)	115 (25%)	84 (22%)	87 (22%)	
Peak stimulated GH (µg/l)^a^	2.7 ± 4.8	2.5 ± 2.5	2.2 ± 2.3	0.223
Lipid concentrations				
Total cholesterol (mg/dl)	210.0 ± 52.9	208.5 ± 47.8	212.5 ± 42.0	0.561
LDL-cholesterol (mg/dl)	131.7 ± 46.7	130.2 ± 39.3	131.4 ± 38.4	0.918
HDL-cholesterol (mg/dl)	50.1 ± 15.4	49.9 ± 14.1	51.4 ± 17.3	0.453
Triglycerides (mg/dl)	151.0 ± 98.0	150.8 ± 101.1	141.0 ± 82.6	0.346
Framingham risk index	8.09 ± 7.57	7.44 ± 7.36	7.42 ± 7.05	0.493
IGF-I concentration (µg/l)	117.1 ± 81.4	106.7 ± 76.9	112.6 ± 86.9	0.433
IGF-I SDS	− 2.28 ± 2.34	− 2.27 ± 2.14	− 2.73 ± 2.73	0.484
Quality of life *Z*-score^b^	− 1.20 ± 1.45	− 1.07 ± 1.46	− 1.16 ± 1.24	0.777

Data show mean ± SD or number of patients (% of total)

*GH* growth hormone, *GHRH* GH releasing hormone, *LDL* low-density lipoprotein, *HDL* high-density lipoprotein, *IGF-I* insulin-like growth factor-I, *SDS* standard deviation scores

^a^GHRH + arginine test only, group A *n * = 274, group B *n *= 262, group C *n *= 290

^b^Group A *n *= 95, group B *n * = 121, group C *n *= 90

There was a significant difference between the groups in the type of stimulation test used for diagnosis of GH deficiency (Table 2). In group C, with the longest duration of GH treatment, the GHRH + arginine test was used more frequently, and tests using either GHRH or arginine alone and the insulin tolerance test were used less frequently than for the other groups. The difference was significant overall and in AO, but not CO, patients and in male, but not female, patients.

**Table 2:** GH stimulation tests used for GH deficiency diagnosis, by frequency of use

	Group A	Group B	Group C	*P* value
All patients	*N *= 424	*N *= 365	*N *= 378	0.008
GHRH + arginine test	275 (65%)	263 (72%)	292 (77%)	
Insulin tolerance test	60 (14%)	47 (13%)	37 (10%)	
GHRH test	44 (10%)	19 (5%)	25 (7%)	
Arginine test	33 (8%)	19 (5%)	24 (6%)	
Adult onset patients	*N *= 323	*N *= 286	*N *= 293	0.031
GHRH + arginine test	224 (69%)	218 (76%)	229 (78%)	
Insulin tolerance test	36 (11%)	30 (10%)	25 (9%)	
GHRH test	37 (12%)	19 (7%)	18 (6%)	
Arginine test	24 (7%)	10 (3%)	17 (6%)	
Childhood onset patients	*N *= 101	*N *= 79	*N *= 85	0.064
GHRH + arginine test	51 (50%)	45 (57%)	63 (74%)	
Insulin tolerance test	24 (24%)	17 (22%)	12 (14%)	
GHRH test	7 (7%)	0	7 (8%)	
Arginine test	9 (9%)	9 (11%)	7 (8%)	
Male patients	*N *= 240	*N *= 220	*N *= 223	< 0.001
GHRH + arginine test	151 (63%)	150 (68%)	185 (83%)	
Insulin tolerance test	40 (17%)	29 (13%)	18 (8%)	
GHRH test	28 (12%)	13 (6%)	8 (4%)	
Arginine test	18 (8%)	14 (6%)	13 (6%)	
Female patients	*N *= 184	*N *= 145	*N *= 155	0.106
GHRH + arginine test	124 (67%)	113 (78%)	107 (69%)	
Insulin tolerance test	20 (11%)	18 (12%)	19 (12%)	
GHRH test	16 (9%)	6 (4%)	13 (6%)	
Arginine test	15 (8%)	5 (3%)	11 (7%)	

Data show number of patients (% of *N*)

*GH* growth hormone, *GHRH* GH-releasing hormone

The difference between groups for the cause of GH deficiency, as reported by the investigator, was statistically significant for AO patients, although not for patients overall or for CO patients (Table 3); there was also no significant difference when analysed by gender. For the AO patients, there was a higher proportion with a diagnosis of craniopharyngioma and a lower proportion with idiopathic GH deficiency in group C with the longest treatment duration in the study.

**Table 3:** Reported cause of growth hormone deficiency

	Group A	Group B	Group C	*P* value
All patients	*N *= 451	*N *= 387	*N *= 395	0.193
Pituitary adenoma	181 (40%)	144 (37%)	170 (43%)	
Craniopharyngioma	51 (11%)	55 (14%)	60 (15%)	
Idiopathic	62 (14%)	48 (12%)	42 (11%)	
Other tumours	23 (5%)	28 (7%)	21 (5%)	
Other causes	134 (30%)	112 (29%)	102 (26%)	
Adult onset patients	*N *= 336	*N *= 303	*N *= 308	0.028
Pituitary adenoma	177 (53%)	143 (47%)	164 (53%)	
Craniopharyngioma	36 (11%)	36 (12%)	43 (14%)	
Idiopathic	19 (6%)	26 (9%)	10 (3%)	
Other tumours	13 (4%)	24 (8%)	14 (5%)	
Other causes	91 (27%)	74 (24%)	77 (25%)	
Childhood onset patients	*N *= 115	*N *= 84	*N *= 87	0.055
Pituitary adenoma	4 (3%)	1 (1%)	6 (7%)	
Craniopharyngioma	15 (13%)	19 (23%)	17 (20%)	
Idiopathic	43 (37%)	22 (26%)	32 (37%)	
Other tumours	10 (9%)	4 (5%)	7 (8%)	
Other causes	43 (37%)	38 (45%)	25 (29%)	

There was no statistical difference between the groups for mean GH dose at start of treatment, except in female patients where mean dose increased with increasing treatment duration (Table 4). Mean GH dose at the last study visit also did not differ significantly between groups, again, except for female patients where those with longest duration had a higher mean dose than those with shortest duration. The mean change in dose from baseline to last visit was significantly different for patients overall and in AO, but not CO, and in male, but not female, patients. The largest decrease in all subgroups was seen in the group with the longest duration of treatment. GH dose at last visit was significantly affected by age (*P *< 0.001), but the interaction was not significant (*P *= 0.063) indicating that the age effect was similar in each group.

**Table 4:** Growth hormone (GH) dose (µg/kg/day) at start of treatment, at last visit, and the change from start to last visit for all patients with GH deficiency, and for GH deficiency group by time of onset

	Group A	Group B	Group C	*P* value
All patients	*N *= 239	*N *= 335	*N *= 373	
Treatment start	5.2 (4.6 to 5.7)	5.5 (5.1 to 6.0)	5.6 (5.3 to 6.0)	0.328
Last visit	5.0 (4.4 to 5.5)	5.9 (5.2 to 6.5)	5.3 (4.9 to 5.8)	0.076
Change to last visit	− 0.1 (− 0.3 to 0.1)	0.2 (− 0.1 to 0.6)	− 0.4 (− 0.7 to − 0.2)	0.004
Adult onset patients	*N *= 173	*N *= 261	*N *= 289	
Treatment start	4.2 (3.8 to 4.7)	4.7 (4.3 to 5.1)	5.0 (4.6 to 5.4)	0.064
Last visit	4.2 (3.7 to 4.7)	5.1 (4.3 to 5.8)	4.6 (4.2 to 5.1)	0.149
Change to last visit	− 0.1 (− 0.3 to 0.2)	0.3 (− 0.2 to 0.7)	− 0.4 (− 0.7 to − 0.1)	0.017
Childhood onset patients	*N *= 66	*N *= 74	*N *= 84	
Treatment start	7.5 (6.1 to 8.9)	8.5 (7.2 to 9.9)	7.9 (7.1 to 8.7)	0.500
Last visit	7.1 (5.8 to 8.4)	8.7 (7.3 to 10.0)	7.7 (6.8 to 8.6)	0.180
Change to last visit	− 0.1 (− 0.3 to 0.1)	0.1 (− 0.4 to 0.5)	− 0.4 (− 0.9 to 0.0)	0.226
Male patients	*N *= 133	*N *= 205	*N *= 221	
Treatment start	5.0 (4.2 to 5.9)	5.0 (4.4 to 5.5)	4.9 (4.5 to 5.3)	0.902
Last visit	4.7 (3.9 to 5.4)	5.2 (4.3 to 6.1)	4.4 (4.0 to 4.7)	0.181
Change to last visit	− 0.3 (− 0.5 to 0.0)	0.2 (− 0.4 to 0.7)	− 0.6 (− 0.9 to − 0.3)	0.030
Female patients	*N *= 106	*N *= 130	*N *= 152	
Treatment start	5.3 (4.6 to 6.0)	6.4 (5.7 to 7.2)	6.8 (6.1 to 7.5)	0.015
Last visit	5.4 (4.6 to 6.2)	6.9 (6.0 to 7.7)	6.7 (5.9 to 7.6)	0.037
Change to last visit	0.2 (− 0.2 to 0.5)	0.3 (0.0 to 0.5)	− 0.2 (− 0.6 to 0.2)	0.113

Data show mean (95% CI)

The increase in mean BMI from baseline to last visit differed significantly by treatment duration, increasing more in patients with the longest duration of treatment (Table 5). BMI at last visit was not significantly affected by age at last visit, either as a direct effect (*P *= 0.093) or interaction (*P *= 0.953). The significant difference between the groups for increase in mean BMI was seen overall and in CO patients, but not AO patients or in the by-gender analysis. The increase in mean IGF-I SDS at last visit was greater in group C with the longest duration of treatment. The difference between the groups for IGF-I SDS increase was significant overall and for female patients, but not for the other categories. Age at last visit had an effect on IGF-I SDS at last visit (*P *= 0.003), while the interaction with group effect was not significant (*P *= 0.300).

**Table 5:** Changes from baseline to last visit for body mass index, IGF-I SDS, lipid concentrations and Framingham cardiovascular risk index

	Group A	Group B	Group C	*P* value
Body mass index (kg/m^2^)				
All patients	0.1 (− 0.1 to 0.3)	0.4 (0.1 to 0.7)	0.6 (0.3 to 1.0)	0.013
Adult onset patients	0.1 (− 0.2 to 0.2)	0.2 (0.0 to 0.5)	0.3 (0.0 to 0.7)	0.319
Childhood onset patients	0.3 (− 0.1 to 0.7)	1.0 (0.3 to 1.6)	1.7 (1.0 to 2.4)	0.003
Male patients	0.1 (− 0.1 to 0.3)	0.2 (− 0.1 to 0.5)	0.5 (0.2 to 0.9)	0.102
Female patients	0.2 (− 0.1 to 0.4)	0.7 (0.3 to 1.2)	0.8 (0.2 to 1.4)	0.071
IGF-I SDS				
All patients	1.3 (0.8 to 1.8)	1.1 (0.6 to 1.7)	2.2 (1.4 to 2.9)	0.039
Adult onset patients	1.5 (0.9 to 2.1)	1.1 (0.5 to 1.6)	1.8 (1.2 to 2.5)	0.173
Childhood onset patients	0.7 (− 0.5 to 1.8)	1.7 (− 0.5 to 3.8)	4.0 (0.5 to 7.5)	0.063
Male patients	1.7 (1.0 to 2.4)	0.9 (0.3 to 1.5)	1.9 (1.0 to 2.8)	0.108
Female patients	0.7 (0.1 to 1.3)	1.4 (0.5 to 2.4)	2.7 (1.2 to 4.1)	0.039
Total cholesterol (mg/dl)				
All patients	− 1.6 (− 4.8 to 1.5)	− 1.1 (− 6.4 to 4.3)	− 10.1 (− 15.0 to − 5.2)	0.008
Adult onset patients	− 1.1 (− 4.6 to 2.5)	− 3.8 (− 9.9 to 2.4)	− 12.8 (− 18.4 to − 7.1)	0.005
Childhood onset patients	− 3.4 (− 10.4 to 3.5)	10.0 (− 0.8 to 20.8)	− 0.6 (− 10.3 to 9.2)	0.109
Male patients	− 2.7 (− 6.6 to 1.3)	0.8 (− 5.9 to 7.4)	− 15.3 (− 21.8 to − 8.9)	< 0.001
Female patients	− 0.3 (− 5.4 to 4.8)	− 3.8 (− 12.9 to 5.2)	− 1.9 (− 9.3 to 5.5)	0.790
LDL cholesterol concentration (mg/dl)				
All patients	− 2.4 (− 6.0 to 1.2)	− 0.5 (−5.9 to 4.9)	− 10.3 (− 15.3 to − 5.4)	0.008
Adult onset patients	− 1.6 (− 5.2 to 2.1)	− 2.8 (− 8.8 to 3.3)	− 13.1 (− 18.6 to − 7.6)	0.003
Childhood onset patients	− 5.7 (− 16.4 to 5.1)	10.4 (− 1.6 to 22.4)	0.7 (− 10.0 to 11.3)	0.142
Male patients	− 3.3 (− 8.4 to 1.8)	1.7 (− 5.4 to 8.8)	− 12.3 (− 18.9 to − 5.7)	0.007
Female patients	− 1.2 (− 6.4 to 3.9)	− 3.8 (− 12.2 to 4.7)	− 7.0 (− 14.2 to 0.2)	0.511
Triglycerides (mg/dl)				
All patients	− 2.0 (− 9.2 to 5.2)	− 8.5 (− 18.6 to 1.6)	6.7 (− 3.8 to 17.1)	0.078
Adult onset patients	− 5.5 (− 13.8 to 2.9)	− 6.1 (− 16.7 to 4.4)	− 0.0 (− 11.1 to 11.1)	0.650
Childhood onset patients	9.2 (− 5.2 to 23.5)	− 20.6 (− 51.2 to 10.0)	30.3 (4.2 to 56.4)	0.016
Male patients	− 7.3 (− 17.8 to 3.2)	− 10.6 (− 26.2 to 4.9)	3.2 (− 11.7 to 18.2)	0.340
Female patients	5.0 (− 4.5 to 14.4)	− 5.3 (− 15.5 to 4.8)	12.1 (− 0.9 to 25.1)	0.088
Framingham cardiovascular risk index				
All patients	− 0.24 (− 0.66 to 0.18)	1.27 (0.87 to 1.67)	1.42 (1.03 to 1.81)	< 0.001
Adult onset patients	− 0.10 (− 0.52 to 0.33)	1.17 (0.76 to 1.58)	1.57 (1.16 to 1.99)	< 0.001
Childhood onset patients	− 0.62 (− 1.70 to 0.46)	1.67 (0.51 to 2.83)	0.79 (− 0.25 to 1.82)	0.027
Male patients	− 0.66 (− 1.12 to − 0.21)	1.42 (0.91 to 1.94)	1.45 (1.00 to 1.90)	< 0.001
Female patients	0.37 (− 0.41 to 1.14)	1.04 (0.41 to 1.66)	1.36 (0.63 to 2.09)	0.206

Data show mean (95% CI)

*LDL* low-density lipoprotein, *IGF-I* insulin-like growth factor-I, *SDS* standard deviation scores, *CI* confidence interval

There was a decrease from baseline to last visit in mean total cholesterol and in mean LDL-cholesterol concentrations in group C with the longest duration of treatment; for both concentrations, 95% CI excluded 0, indicating a significant effect, for patients overall and AO and male patients (Table 5). The interaction of age at last visit with treatment duration difference was significant for total cholesterol (*P* < 0.001) and LDL-cholesterol (*P* = 0.017) concentrations at last visit, indicating that the effect of age differed between the groups. Changes in triglyceride concentrations were very variable, with wide 95% CI, and showed little effect of GH treatment duration except for CO patients where the longest duration group showed an increase in concentration. There was a significant interaction of age with treatment duration on triglyceride concentrations at last visit (*P *= 0.019). No significant between-group differences were seen for changes in HDL-cholesterol concentration.

The mean Framingham cardiovascular risk index increased to a greater extent in the groups with longer duration of GH treatment, with no overlap of 95% CI values for group A (shortest duration) and group C (longest duration), for patients overall and AO and male patients. At last study visit (Table 5), there was a significant effect of treatment duration for patients overall and by category of AO and CO GH deficiency and in male patients, though not in female patients. There was no significant effect of age at last visit on change in Framingham cardiovascular risk at last visit (*P *= 0.593) and interaction of age with GH treatment duration was not significant (*P *= 0.064).

The QLS-H questionnaire was completed by only a limited number of patients. At baseline, the mean and 95% CI for the quality of life *Z*-score was below 0 for each group [group A − 1.20 (95% CI − 1.49 to − 0.90); group B −1.07 (95% CI − 1.33 to − 0.81); group C − 1.16 (95% CI − 1.42 to − 0.90)], with no significant difference between the treatment duration groups (*P *= 0.777). There were insufficient numbers of patients with available data to evaluate changes from baseline.

## Discussion

Patients with GH deficiency, whether starting during adulthood or with onset during childhood and continuing in adulthood, require GH treatment throughout life to correct metabolic abnormalities. Our analysis of metabolic parameters in adult patients with GH deficiency in Italy showed effects of duration of GH treatment on IGF-I SDS, BMI, lipids and Framingham cardiovascular risk. The analysis also indicated differences in the GH stimulation tests used and the diagnosed cause of the GH deficiency.

In Italy, the GHRH + arginine test was used for approximately 70% of patients, in contrast to the global data from HypoCCS where the test was used in less than a quarter of the patients [11]. In Italy, a higher proportion of patients in the longest GH treatment duration group received the GHRH + arginine test, whereas a lower proportion of patients received other tests. This could indicate that physicians were more confident about long-term GH replacement for patients diagnosed as GH-deficient using the more sensitive and accurate GHRH + arginine test [16]. This was particularly noticeable for AO patients and was consistent with the less-frequent diagnosis of idiopathic GH deficiency and more-frequent diagnosis of craniopharyngioma in the AO patients in the longest duration of treatment group. However, it may also relate to changes over time in GH stimulation tests and diagnoses that were observed previously for patients entered into HypoCCS between 1996 and 2005 [11].

GH dose for patients overall was not significantly different between treatment duration groups at the start of treatment, but was decreased from baseline to last study visit to a greater extent in the group with the longest treatment duration. In the patients with the longest GH treatment duration, the 95% CI did not include zero, indicating a significant decrease in dose. Because the aim was to assess changes according to duration of GH treatment, the whole cohort including all three groups was not analysed; however, a previous analysis of the patients, on the basis of severity of GH deficiency, indicated that the dose increased over the first 5 years and then fluctuated [12], suggesting that for the whole cohort the dose decrease in the longer duration group was balanced by an increase in the shorter duration group. Mean doses in female patients were higher than in male patients at treatment start and last visit, with no overlap in 95% CI for the two longer duration groups, and in female patients no significant difference was seen between groups for change to last visit. This was consistent with the need for a higher GH dose in female patients and indicated that endocrinologists in Italy involved in HypoCCS were aware of the pathophysiological issue that female patients tend to be more resistant to GH than male patients [17–20]. Mean doses in patients with CO GH deficiency were higher than in those with AO GH deficiency, consistent with the requirement of a higher dose in patients at a younger age to optimise body proportions [21]. GH dose in overall patients with the longest duration of treatment significantly decreased from baseline to last visit, as indicated by 95% CI values that did not include 0. This reduction in dose could reflect a decreased requirement based on the physiological decrease of IGF-I with age [22, 23]. A significant direct effect of age at last visit was observed for GH dose at last visit, suggesting that the decrease in dose could possibly be more evident in patients with longer term treatment.

IGF-I SDS was increased to a greater extent in patients with longer duration of GH treatment overall, although this was associated more with female and CO patients reflecting their higher GH doses. Decreasing IGF-I with ageing has been shown in some studies to be associated with increased atherosclerosis and cardiovascular disease events [24–26], and it was reported that this was related to components of the metabolic syndrome [26]. An increase in BMI in the patients with the longest duration of treatment was observed, with a significant difference between duration groups. This was seen for patients overall, but was particularly apparent in patients with CO GH deficiency. While BMI generally tends to increase with age in the general population [27–29], no significant effect of age at last visit or interaction with duration of treatment was found; however, adult GH-deficient patients have a higher BMI at a younger age with no change with increasing age [29] and when treated with GH, an increased BMI was only seen after 7 years or more [9].

Significant decreases in total and LDL-cholesterol concentrations occurred in the group of patients with the longest duration of GH treatment; this may indicate persistent effects of GH treatment on these parameters. The 95% CI values for decreases in both total cholesterol and LDL-cholesterol excluded 0 in the group with the longest duration of GH treatment for patients overall, AO patients and male patients. GH treatment of adult patients has been shown in several studies to decrease total and LDL-cholesterol levels [1, 4, 8, 21]. In contrast to total and LDL-cholesterol, triglyceride concentrations appeared to increase in patients with longest duration of GH treatment; however, this was primarily due to an increase in the patients with CO GH deficiency. The results for triglycerides were in accordance with studies that generally show no effect of GH treatment on triglyceride concentrations [8, 10] and may simply reflect an increase with ageing.

Despite the decreased total and LDL-cholesterol concentrations in the group of patients with longest duration of GH therapy, the Framingham cardiovascular risk index increased in these patients as expected due to the progressive ageing of patients. The significant difference between GH treatment duration groups was seen overall, in patients with AO GH deficiency and in male patients. However, the increase in the Framingham cardiovascular risk index may be the result of the increased age of patients in the longest duration group. Total cholesterol was significantly decreased and HDL-cholesterol was not significantly changed, while LDL-cholesterol was significantly increased but not included in Framingham score calculation. Additionally, systolic blood pressure was not analysed but considered unlikely to change, and the proportion of smokers was similar during the study. Therefore, it is clear that patients’ age at last visit is likely to have had a major impact on Framingham cardiovascular risk without having a significant effect overall. This would suggest that GH, at the dose used, could not control all the factors involved in cardiovascular risk and highlights the importance of continued observation of all factors associated with cardiovascular risk during long-term GH replacement in adult patients.

The strength of this study lies in providing data on GH therapy according to duration of follow-up that has been obtained from daily clinical practice.

In conclusion, in a real-life setting such as the Italian HypoCCS study, increased IGF-I SDS, decreased LDL-cholesterol, more robust diagnostic tests and more severe causes of hypopituitarism (i.e. craniopharyngioma) are associated with a longer term duration of GH therapy. Thus, the presence of such surrogate markers that help to give confidence in a diagnosis of GH deficiency predicts a longer duration of treatment.
